# Single-cell transcriptome reveals potential mechanisms for gout in children

**DOI:** 10.3389/fimmu.2025.1577109

**Published:** 2025-04-30

**Authors:** Shengyou Yu, Ren Qi, Liang Xiao, YiHui Huang, Li Yu

**Affiliations:** ^1^ Department of Pediatrics, Guangzhou First People’s Hospital, South China University of Technology, Guangzhou, Guangdong, China; ^2^ Department of Rheumatology and Immunology, Guangzhou Women and Children’s Medical Center, Guangzhou Medical University, Guangzhou, Guangdong, China; ^3^ Department of Pediatrics, Guangzhou Red Cross Hospital of Jinan University, Guangzhou, Guangdong, China

**Keywords:** pediatric gout, single-cell RNA sequencing, CD14+ monocytes, DN T cells, inflammatory mechanisms children

## Abstract

**Objective:**

Pediatric gout is a condition that differs from traditional adult gout and has attracted significant attention. This study aims to explore the molecular mechanisms underlying pediatric gout.

**Methods:**

We analyzed peripheral blood samples from pediatric gout patients and healthy controls using single-cell RNA sequencing (scRNA-seq). Statistical tests were employed to analyze the data and identify significant differences between the groups.

**Results:**

Our findings revealed that CD14+ monocytes and DN T cells play crucial roles in pediatric gout. CD14+ monocytes are essential for recognizing and phagocytosing monosodium urate (MSU) crystals, triggering inflammation. DN T cells may be involved in the adaptive immune response within gouty joints. These cells not only contribute to the inflammatory response but also interact with other immune cells, amplifying the inflammatory cascade. Comparative analysis with adult gout studies highlighted both similarities and differences in cellular and molecular mechanisms between children and adults. The CD14+ monocytes may be interact with other immune cells through the TNF-α/NF-κB signaling pathway. Targeting this pathway may offer therapeutic potential for managing pediatric gout.

**Conclusion:**

The results provide a foundation for new diagnostic markers and therapeutic targets for pediatric gout. They also pave the way for future research and the development of targeted therapies that can effectively manage and potentially prevent the debilitating effects of gout in children. Understanding the unique molecular mechanisms in pediatric gout could influence treatment strategies and improve patient outcomes.

## Introduction

Gout, historically considered a disease predominantly affecting adults, has seen a concerning rise in pediatric incidence rates in recent years. This trend is alarming as pediatric gout is linked to a range of adverse health outcomes, including acute inflammatory arthritis, chronic tophaceous gout, and potentially long-term complications such as kidney disease (1–4). The impact of the disease on quality of life and the associated healthcare burden underscore the urgency for a deeper understanding of its pathogenesis in children.

With the deepening of immunological research, the important role of immune-inflammatory responses in the pathogenesis of gouty arthritis has gained increasing attention. however, their roles in gout are unclear. the detailed role of monosodium urate crystals in the pathogenesis of gout still needs further investigation. In addition to resident immune cells, disarrangement of innate immune signaling in tissue cells also contributes to the pathogenesis of metabolic diseases (5–7). These interactions activate immune cells, including macrophages, monocytes, lymphocytes, and neutrophils, which secrete pro-inflammatory cytokines such as tumor necrosis factor-α (TNF-α), interleukin (IL)-1β, IL-8, IL-10, IL-17, and IL-37. These cytokines induce a series of cascade inflammatory amplification reactions (8–11). Importantly, the etiology of gout in children differs significantly from that in adults. Pediatric gout encompasses a broader spectrum of secondary factors and hereditary conditions. For example, conditions such as leukemia, lymphoma, sickle cell anemia, and the use of cytotoxic chemotherapy can lead to secondary hyperuricemia and gout in children. Additionally, inborn errors of purine metabolism can result in overproduction of uric acid, highlighting the genetic component in pediatric gout (12–15). The heterogeneity in disease presentation and the influence of genetic and acquired factors complicate diagnosis and treatment, leading to a scarcity of evidence-based therapeutic approaches tailored to the pediatric population. Current treatment paradigms for gout have been largely informed by adult studies (16–18), and thus, current therapeutic approaches may not be optimally effective or safe for children. There is an urgent need to delineate the unique pathophysiological pathways underlying gout in children to guide tailored therapeutic strategies.

Single-cell RNA sequencing (scRNA-seq) offers a powerful tool for in-depth dissection of the cellular and molecular mechanisms of pediatric gout. By analyzing the transcriptomic profiles of individual cells, we can identify distinct cell populations and their functional states in children with gout, potentially revealing novel biomarkers, uncovering cell-specific dysregulations, and elucidating the molecular mechanisms of pediatric gout. Based on this, the present study employs scRNA-seq to examine immune cell subsets in pediatric gout and identify cellular and molecular signatures that distinguish pediatric gout from adult gout. Our findings are expected to fill the knowledge gap in pediatric gout pathogenesis and lay the foundation for the development of more effective and personalized therapeutic strategies.

## Methods

### Ethics

Informed consents were obtained from all children and their parents, and the study was approved by the Research Ethics Committee of Guangzhou First People’s Hospital, South China University of Technology, China(K-2024-129-02). Both the Declaration of Helsinki and the Good Clinical Practice Guidelines were followed and informed consent granted by all participants.

### Patient recruitment

In this study, We included three healthy children and three pediatric gout patients, children with gout fulfilled the classification criteria of gouty arthritis (1). All of the cases in the discovery and validation stages were recruited using the same diagnostic criteria. The healthy controls were attained via site survey. Inclusion Criteria: Approved by the ethics committee; Under 18 years of age; Mentally sound and conscious; Signed informed consent form. Exclusion Criteria: Gout patients caused by other reasons; Poor compliance; Those with respiratory failure, heart failure, and malignant tumors; Presence of depression, schizophrenia; Severe joint stiffness, deformity; Those with organic diseases; Those with serious infections. We collected basic patient information including age, uric acid level, glomerular filtration rate, and disease duration. Peripheral blood was obtained from three children with gout and three normal children, and single‐cell suspension was immediately prepared and subjected to scRNA‐seq analysis. The sample collection and the clinical information regarding the subjects were undertaken following informed consent and approval by the relevant ethics review board at the Guangzhou First People’s Hospital in accordance with the tenets of the Declaration of Helsinki. All children and their parents consented to having their peripheral blood used for research purposes.

## Data analysis

### Quality control and preprocessing

To ensure high-quality data, raw sequencing reads were first assessed using FastQC (v0.11.9) to examine base quality scores, GC content, and adapter contamination. Adapter trimming and low-quality base filtering were then performed using Trimmomatic (v0.39) with the following parameters: LEADING: 3 TRAILING: 3 SLIDINGWINDOW: 4:15 MINLEN: 36. The cleaned reads were re-evaluated with FastQC to verify improvement in data quality. The average sequencing depth per cell exceeded 50,000 reads, which meets standard thresholds for immune profiling with 10x Genomics protocols.

### Alignment, barcode assignment, and UMI counting

Clean reads were aligned to the human reference genome (GRCh38, release 2020-A) using Cell Ranger (v6.1.2), which utilizes the STAR aligner. Cell Ranger mapped reads to the genome and classified them as exonic, intronic, or intergenic, only retaining reads that were confidently aligned to the transcriptome (MAPQ≥255) for unique molecular identifier (UMI) counting. To identify true cells, Cell Ranger uses a dynamic thresholding algorithm based on UMI count distribution. Specifically, we set the expected cell number (expect-cells) to 3,000, and barcodes with UMI counts above 1/10 of the 99th percentile UMI count (denoted as m/10) were retained. This strategy filters out ambient RNA and empty droplets while ensuring accurate cell identification. After filtering, we obtained a total of 16,728 high-quality single cells across all six samples.

### Batch effect correction and data integration

Since multiple samples were processed, batch effects may confound biological variability. To address this, we employed the Harmony algorithm (R package: harmony, v1.0), integrated within the Seurat v4.3.0 workflow. Harmony was applied on the top 30 principal components to align the dataset across patients and controls, minimizing technical artifacts while preserving biological signals.

### Dimensionality reduction and cell clustering

Normalized gene expression data were reduced in dimensionality using Principal Component Analysis (PCA). We selected the top 30 principal components based on the elbow plot and JackStraw analysis for downstream clustering. For visualization, PCA-reduced data were embedded in two dimensions using t-SNE (t-distributed Stochastic Neighbor Embedding) with a perplexity of 30. Unsupervised clustering was performed using Louvain graph-based clustering with a resolution parameter of 0.8 (in FindClusters()), balancing cluster granularity and biological interpretability. This clustering approach groups cells with similar expression profiles, facilitating cell-type identification.

### Cell-type annotation

Cell types were assigned based on canonical marker genes (e.g., CD3D for T cells, CD14 for monocytes, MS4A1 for B cells) using Seurat’s FindAllMarkers and validated by comparison to published immune cell reference atlases.

### Differential gene expression analysis

To identify genes differentially expressed between disease and control samples within each cell type, we used MAST (Model-based Analysis of Single-cell Transcriptomics), which is tailored for single-cell data with dropout effects. Statistical significance was defined as: |log2 Fold Change| ≥ 0.25; Adjusted p-value (FDR) < 0.05, corrected via Benjamini-Hochberg method. Top differentially expressed genes (DEGs) were visualized using volcano plots and heatmaps. Notably, CD14+ monocytes in gout patients showed significant upregulation of IL1B, CXCL2, and downregulation of LYZ, reflecting altered innate immune responses.

### Functional enrichment analysis (GO and KEGG)

To understand the biological functions and pathways of DEGs, we performed Gene Ontology (GO) and Kyoto Encyclopedia of Genes and Genomes (KEGG) enrichment analyses using the clusterProfiler R package (v4.8.1). GO enrichment focused on the Biological Process (BP) domain, and pathway analysis was based on KEGG 2021 database. Gene set enrichment significance was defined by: Adjusted p-value < 0.05; Gene length bias corrected using the enricher function. Enrichment results highlighted the involvement of pathways such as TNF signaling via NF-κB, endocytosis, autophagy regulation, and histone modification in pediatric gout pathogenesis.

## Results

### Validation of abnormal cell types

**Figure 1:**
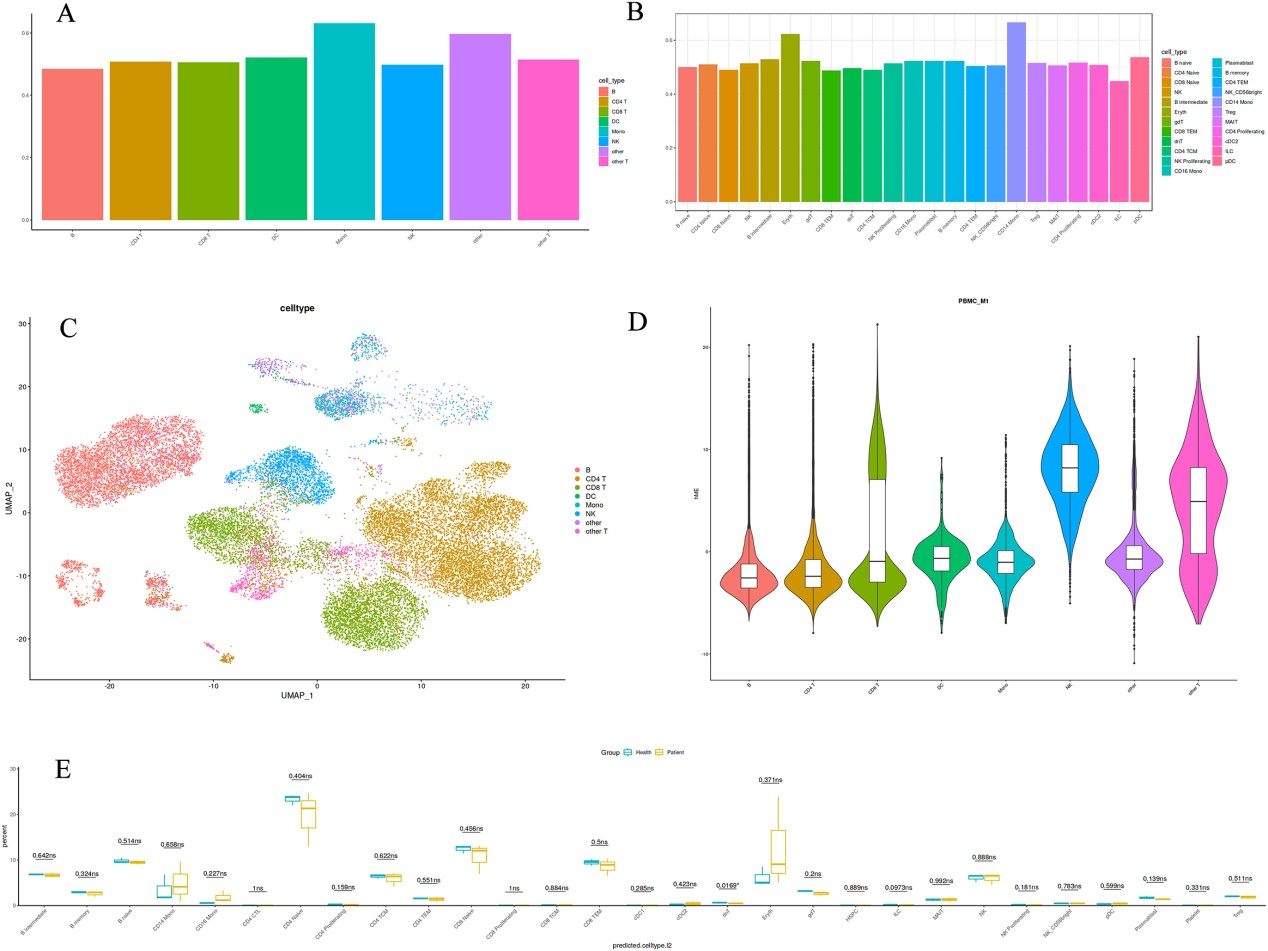
Immunological landscape of immune cells from healthy children and pediatric gout patients. **(A)**: The large class annotation highlights the prominence of monocytes; **(B)**: The refined annotation emphasizes the significance of CD14+ Mono cells; **(C)**: A single-cell type map for peripheral blood detection; **(D)**: A violin plot depicting the distribution of a single cell type; **(E)**: A boxplot illustrating the differences between two datasets.

We conducted single-cell RNA sequencing on peripheral blood samples from three healthy children and three pediatric gout patients. After rigorous quality control, the primary cell types identified were B cells, CD4+ T cells, CD8+ T cells, monocytes, and others (**Figures 1A, C, D**). To ascertain which cell types exhibit abnormalities in pediatric gout patients relative to healthy children, we employed a tripartite validation approach. This methodology encompassed comprehensive single-cell RNA sequencing (scRNA-seq) analysis, coupled with algorithmic prioritization using Augur, and comparative cytometric assessments. Our findings consistently indicated a significant deviation in the quantity of CD14+ monocytes between the health and patient cohorts (**Figures 1B, E**). Specifically, the patient group demonstrated a markedly elevated presence of CD14+ monocytes, suggesting a pivotal role for these cells in the pathogenesis of gout in children.

### Differential gene expression analysis

**Figure 2:**
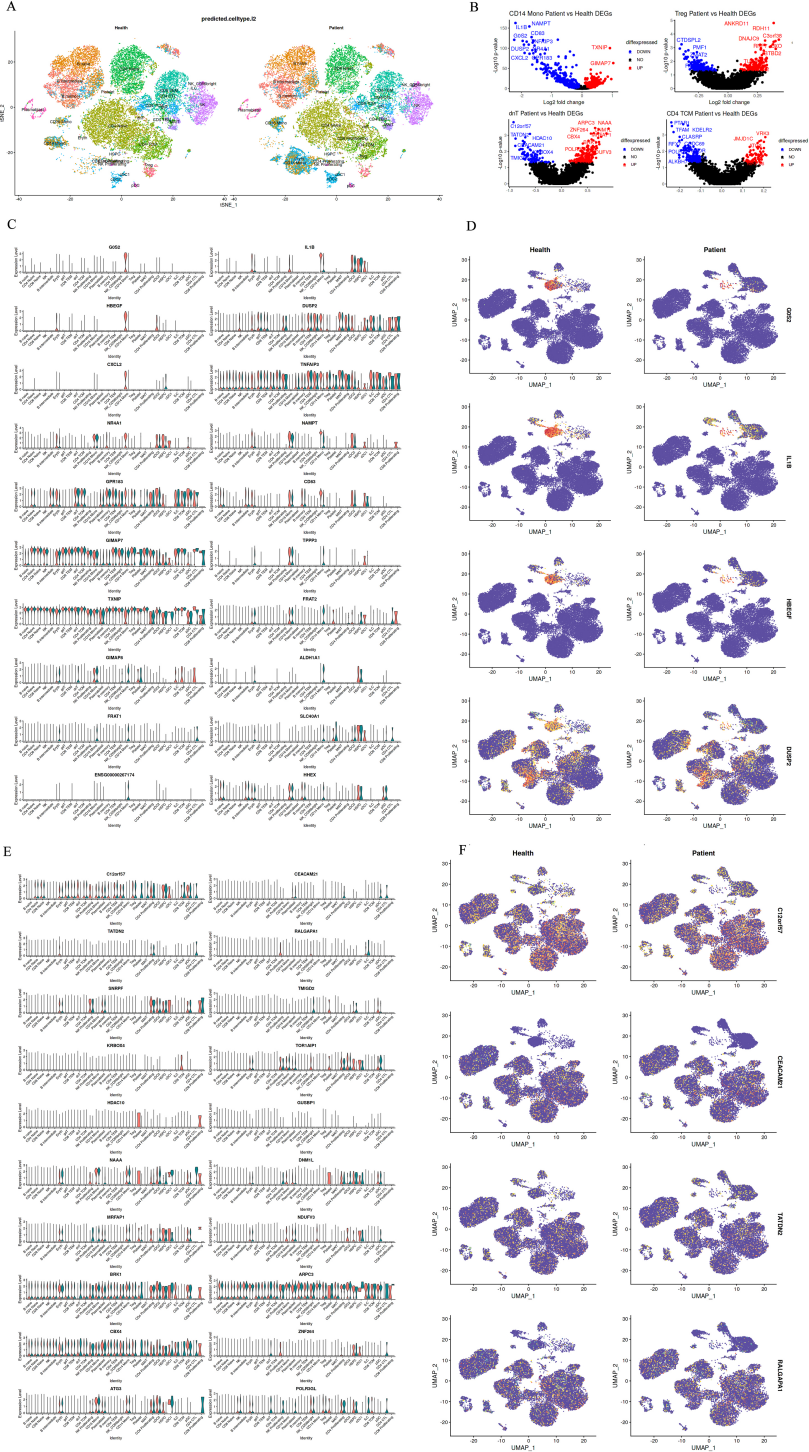
Differential Gene Expression Between Groups of Differential Subsets. **(A)**: Top 10 differentially expressed genes in CD14+ Mono cells between healthy children and pediatric gout patients (red indicates normal expression, blue indicates expression in patients with disease); **(B)**: Top 10 genes with differential up- and down-regulation in CD14+ and dnT cell differential analysis; **(C, D)**: Expression distribution of differentially expressed genes in CD14 Mono cells between healthy and diseased cell types; **(E, F)**: Expression distribution of differentially expressed genes in dnT cells between healthy and diseased cell types.

Visual representation of the top 10 differentially expressed genes in CD14+ monocytes and dnT cells further elucidated the molecular discrepancies between health and disease states (**Figures 2A, B**). Heatmaps depicted the contrasting expression patterns of these genes, with red signifying higher expression in healthy controls and blue indicating elevated expression in gout patients. Notably, the gene expression landscapes of CD14+ monocytes and dnT cells were starkly different between the two groups, underscoring the heterogeneity of cellular responses in pediatric gout.

For CD14+ monocytes, genes like”IL1B”and “CXCL2”were prominently upregulated in patients, aligning with heightened pro-inflammatory activity. In contrast, genes such as “CD14” and “LYZ”showed reduced expression, possibly impacting the cells’ ability to respond to microbial stimuli and process antigens (**Figures 2C, D**). For dnT cells, the top differentially expressed genes included”TATDN2”and “CEACAM21”, suggesting a potential skew towards cytotoxic activity in the patient cohort (**Figures 2E, F**). These findings, when juxtaposed with the cellular composition data, underscore the complexity of immune dysregulation in pediatric gout. The observed shifts in gene expression profiles within CD14+ monocytes and dnT cells provide a molecular basis for the clinical manifestations of gout in children and may guide the development of targeted immunomodulatory therapies.

### Gene function enrichment analysis

**Figure 3:**
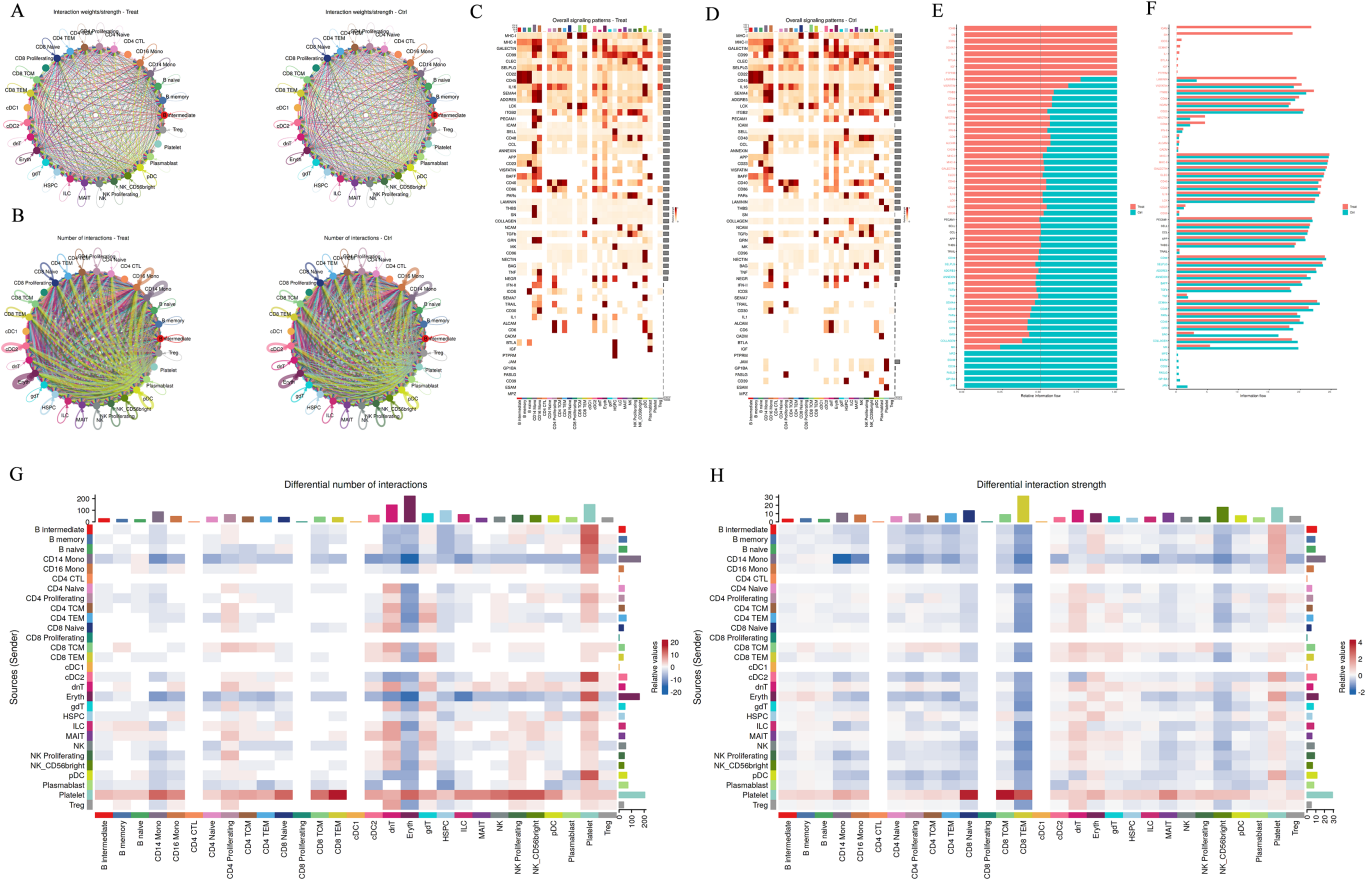
Cell–Cell Interaction Signaling. **(A, B)**: Differential number of interactions or interaction strength among different cell populations; **(C, D)**: The analysis summarizes information from both outgoing and incoming signaling. **(E-H)**: Comparison of the overall information flow for each signaling pathway. Significant signaling pathways were ranked based on differences in the overall information flow within the inferred networks between groups. The top signaling pathways colored red are enriched in WT, while those colored green are enriched in the Mut.

Gene set enrichment analysis (GSEA) of CD14+ monocytes from pediatric gout patients and healthy children provided novel insights into the molecular signaling pathways and mechanisms underlying pediatric gout, potentially paving the way for the development of new therapeutic strategies (**Figures 3A–D**). The analysis revealed that the top five enriched gene sets were associated with monocyte response to lipopolysaccharide (LPS) treatment, notably including the TNF-α signaling pathway through NF-κB. Interestingly, patients exhibited negative normalized enrichment scores (NES), contrasting with the positive NES observed in healthy children. This inverse expression pattern in gout patients suggests that alterations in the TNF-α/NF-κB signaling pathway could serve as a key marker for differentiating between these two conditions.

KEGG analysis of CD14+ monocytes identified several upregulated pathways, such as endocytosis, Salmonella infection, and protein processing in the endoplasmic reticulum. In contrast, downregulated pathways included those related to the ribosome and various neurodegenerative diseases. GO analysis further highlighted upregulated biological processes like proteasome-dependent protein degradation, histone modification, and autophagy regulation, while downregulated processes included cytoplasmic translation and ubiquitin-dependent proteasome-mediated protein degradation (**Figures 3E–H**). Collectively, these results underscore the significant role of protein modification processes in the function of CD14+ monocytes. These findings may guide the activation or inhibition of related signal transduction pathways for early diagnosis and targeted treatment of pediatric gout.

## Discussion

The increasing incidence of gout in pediatric populations represents a significant shift from traditional demographics, where the disease was predominantly observed in adults. This trend is alarming, given the potential for long-term morbidity, including joint damage and renal impairment, which can severely impact a child’s quality of life. The dearth of mechanistic insights into pediatric gout, coupled with the absence of tailored therapeutic strategies, underscores the urgent need for comprehensive research. Understanding the unique pathophysiological mechanisms in children is pivotal for developing effective treatment protocols that address the specific needs of this vulnerable population.

With the deepening of immunological research, the important role of immune-inflammatory responses in the pathogenesis of gouty arthritis has gained increasing attention. Studies have shown that monosodium urate crystals can be recognized by pattern recognition receptors on the surface of innate immune cells such as neutrophils, monocytes/macrophages, NK cells, and mast cells, which detect pathogen-associated molecular patterns (PAMPs) and damage-associated molecular patterns (DAMPs) to activate the innate immune response. In addition, the imbalance of T-cell subsets, abnormal expression of specific cytokines, and abnormal humoral immune responses mediated by B cells also play key roles in the development of this disease. Despite this, the detailed role of monosodium urate crystals in the pathogenesis of gout still needs further investigation. In addition to resident immune cells, disarrangement of innate immune signaling in tissue cells also contributes to the pathogenesis of metabolic diseases, however, their roles in gout are unclear. In recent years, many studies have employed scRNA-seq to examine immune cells in the blood or tissues of pediatric populations (19–23); however, there has been no reported use of this technology to investigate its relationship with gout in children. In this study, we collected peripheral blood samples from three healthy children and three pediatric gout patients for scRNA-seq analysis. Our study has unveiled significant insights into the cellular mechanisms underlying pediatric gout, particularly highlighting the roles of CD14+ monocytes and DN T cells. CD14+ monocytes are well-established as key players in the innate immune response, serving as sentinels that detect and respond to pathogens and tissue damage. In the context of gout, these cells are crucial for the recognition and phagocytosis of monosodium urate (MSU) crystals, which trigger inflammation. Our study revealed a significant enrichment of CD14+ monocytes in pediatric gout patients, suggesting that these cells may be hyperactivated in response to MSU crystals. Furthermore, CD14+ monocytes upregulation in pediatric gout may indicate an intensified inflammatory milieu. These cells are pivotal in the production of pro-inflammatory cytokines such as IL-1β, which is a known mediator of gouty inflammation. The upregulation of genes like”IL1B”and”CXCL2”in CD14+ monocytes further supports their involvement in the activation of the NLRP3 inflammasome, a critical pathway in gout pathogenesis. Studies have confirmed that the accumulation of monosodium urate (MSU) crystals in the joint triggers an innate immune response, with the NALP3 inflammasome being central to initiating the cellular inflammatory cascade (24). Once activated, the NLRP3 inflammasome stimulates the secretion of pro-inflammatory cytokines such as IL-1β, which is a key mediator of the characteristic inflammatory cascade in gout (25). The secretion of these cytokines initiates the recruitment of additional immune cells, setting off an inflammatory cascade. This cascade manifests as the hallmark symptoms of gout. In contrast, the downregulation of genes associated with phagocytosis and antigen presentation may impair the ability of these monocytes to clear urate crystals effectively, contributing to the persistence of inflammation. These studies have also implicated monocytes in the disease process, although the specific gene expression profiles and cellular responses may vary between pediatric and adult populations.

The differential expression of genes in DN T cells, another cell type highlighted in our study, suggests a role for these cells in the adaptive immune response in pediatric gout. DN T cells, characterized by the absence of CD4 and CD8 surface markers, are a heterogeneous population with diverse functions, including cytotoxicity and the production of cytokines. Their role in pediatric gout has been less explored, but our study indicates that these cells may be involved in the adaptive immune response in gouty joints. The upregulation of genes like”TATDN2”and “CEACAM21”in DN T cells suggests a potential skew towards cytotoxic activity, which could contribute to tissue damage in gouty joints. This finding is intriguing, as it suggests that DN T cells may play a role in the direct cytotoxicity observed in gouty inflammation, potentially exacerbating joint damage.

Many studies have shown that immune cells secrete cytokines or chemokines in response to hyperuricemia or urate crystal stimulation; however, there are no data related to interactions among these immune cells (8–10, 26–32). In this study, we found that the involvement of CD14+ monocytes and DN T cells in pediatric gout underscores the complexity of the immune response in this disease. These cells not only contribute to the inflammatory response but also interact with other immune cells, potentially amplifying the inflammatory cascade. This interaction may involve the release of cytokines and chemokines that recruit additional immune cells to the site of inflammation, further exacerbating the inflammatory response. GSEA revealed that the top five enriched gene sets were associated with the TNF-α signaling pathway through NF-κB. MSU is recognized by pattern recognition receptors as a damage-associated molecular pattern, stimulating the innate immune system to mount an immune response, and regulating the transcriptional machinery to activate the NF-κB signaling pathway. Consequently, after the free IκB in the cytoplasm is phosphorylated and ubiquitinated by IκB kinase, NF-κB, as a free complex, translocates to the nucleus, leading to the activation of the NLRP3 inflammasome. Once activated, active NLRP3, ASC, and caspase-1 work together to cleave pro-IL-1β and pro-IL-18 into mature IL-1β and IL-18, which are released into the extracellular environment, ultimately leading to exacerbated inflammation and tissue damage. Therefore, the activation of the NF-κB signaling pathway is an important route for inducing inflammation (33, 34). By regulating the NF-κB signaling pathway, inflammation can be suppressed to achieve the effect of preventing and treating gout. Interestingly, patients exhibited negative normalized enrichment scores (NES), contrasting with the positive NES observed in healthy children. This inverse expression pattern in gout patients suggests that alterations in the TNF-α/NF-κB signaling pathway could serve as a key marker for differentiating between these two conditions. Moreover, Comparative analysis with adult gout studies reveals both similarities and differences. While the involvement of monocytes and T cells in gout is a common thread, the specific cellular and molecular mechanisms may differ between children and adults. This could have implications for treatment strategies, suggesting that therapies targeting the innate immune response may be particularly effective in pediatric populations. These results may provide a new diagnostic marker and therapeutic target for children with gout.

The results of our study underscore the critical need to elucidate the mechanisms underlying pediatric gout to inform clinical treatment. The identification of key cell types and their associated molecular pathways provides a foundation for targeted therapeutic interventions. The significant differences in gene expression profiles between CD14+ monocytes and DN T cells in pediatric gout patients compared to healthy controls highlight the complexity of the immune response in this disease. However, the universality and accuracy of the findings are somewhat limited due to the small sample size, lack of longitudinal data, and absence of functional validation experiments. To overcome these limitations, we will expand the sample size, conduct longitudinal studies, and perform functional validation experiments. Additionally, further exploration of therapeutic interventions, investigation of the interplay between genetic and environmental factors, and comparative studies with adult gout will enhance our understanding of the unique molecular mechanisms of pediatric gout and aid in the development of more effective personalized treatment strategies.

In conclusion, our study provides novel insights into the cellular mechanisms of pediatric gout, highlighting the importance of CD14+ monocytes and DN T cells in the disease process. These findings pave the way for future research and may lead to the development of targeted therapies that can effectively manage and potentially prevent the debilitating effects of gout in children.

## Data Availability

The datasets presented in this study can be found in online repositories. The name of the repository and accession number can be found below: BioProject Archive of the Beijing Institute of Genomics (BIG) Data Center, BIG, Chinese Academy of Sciences (http://bigd.big.ac.cn/gsa-human) accession code PRJCA039329.
